# Surgical Management of Compound Odontoma in a Pediatric Patient: A Case Report

**DOI:** 10.7759/cureus.70729

**Published:** 2024-10-02

**Authors:** Sharifah A Alharthi, Fahad A Althobaiti, Yasser M Ibrahim, Hassan O Alansari, Marwa M Alhothali

**Affiliations:** 1 General Dentistry, Umm Al-Qura University, Mecca, SAU; 2 Family Dentistry, Security Forces Hospital, Mecca, SAU; 3 Oral and Maxillofacial Surgery, Security Forces Hospital, Mecca, SAU; 4 General Dentistry, Ibn Sina National College, Jeddah, SAU; 5 Pediatric Dentistry, Security Forces Hospital, Mecca, SAU

**Keywords:** benign tumor, compound odontoma, hamartoma, paediatric patient, surgical management

## Abstract

Odontomas are the most common type of benign odontogenic tumors, representing around 70% of all odontogenic tumors of the jaws. Odontoma is typically present in the first and second decades of life. Morphologically, compound odontomas appear as deposited dental tissues in a pattern that makes a tooth-like structure. Compound odontomas can occur in any area of the jaws; however, the anterior maxilla is the most common location of compound odontomas. In the current report, we aimed to remove the tumor surgically to avoid unwanted consequences of odontomas, such as eruption disturbance, root resorption, tooth malalignment, and cortical bone expansion.

An 11-year-old female patient presented to the pediatric dental clinic in Security Forces Hospital, Makkah, Saudi Arabia, complaining of multiple carious teeth. A routine panoramic radiograph showed a well-defined radiopaque mass surrounded by a thin halo in the right maxillary anterior region. This mass comprised multiple small, tooth-like structures. Upon pulpation, a palatal bulge was detected on the right side of the anterior part of maxilla. Since the patient showed anxious dental behavior and needed comprehensive dental treatment, she was scheduled for surgical removal of the mass under general anesthesia. Tiny tooth-like structures were removed and sent to the histopathological examination to confirm the diagnosis. Follow-up appointments for the patients were scheduled to be after two weeks, one month, and six months. In conclusion, this case highlights the importance of early diagnosis and prompt surgical intervention in managing compound odontomas to avoid the unwanted consequences of the tumor.

## Introduction

Odontomas are the most common type of benign odontogenic tumors, representing around 70% of all odontogenic tumors of the jaws [1]. Odontomas consist of enamel and dentine in combination with amounts of cementum and pulp as they originate from the odontogenic epithelium and ectomesenchyme tissues [2]. The cause of odontoma is unknown, however, it has been thought that local trauma, infection at the site of the lesion, genetic background, family history, and hereditary syndromes such as Gardner syndrome and basal cell nevus syndrome, might play a role in the formation of odontomas [2,3]. Morphologically, odontomas can be classified into two categories: complex odontomas, which are irregular masses containing different types of dental tissues, and compound odontomas, when dental tissues are deposited in a pattern that makes a tooth-like structure. Both types can occur in any area of the jaws; however, the most common location of complex odontomas is the posterior mandible, whereas the anterior maxilla is the most common location of compound odontomas [4,5]. Most odontomas are slow-growing, non-aggressive, clinically asymptomatic, and are often detected on routine radiographic examination [3]. However, early detection and management are critical to avoid unwanted consequences of odontomas, such as unerupted or impacted teeth, root resorption, tooth malalignment, discomfort, and tumor enlargement, which leads to cortical bone expansion [5]. Generally, odontomas are removed surgically [6,7], and if necessary, additional dental treatment for associated complications, such as bone graft or orthodontic intervention, can be considered [5]. The recurrence rate of compound odontoma is low [7]. In the current case, we plan to remove the lesion surgically to avoid the unwanted consequences of odontomas.

## Case presentation

**Figure 1:**
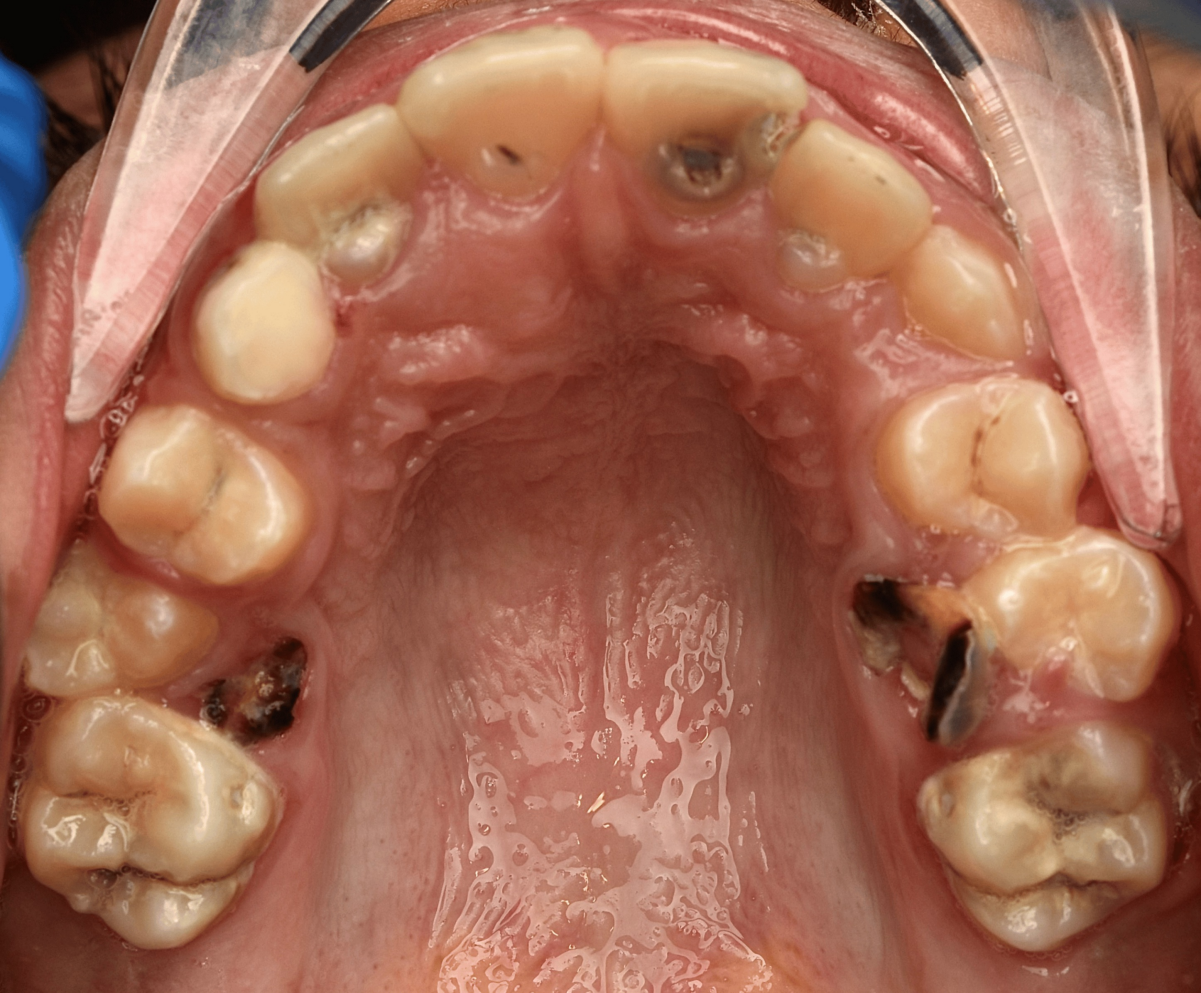
Intraoral view At initial examination, retained primary canine on the right side and a palatal bulge was detected mainly upon palpation.

The current article reports the surgical removal of compound odontoma in a dentally anxious 11-year-old female who attended our dental office complaining of multiple carious teeth. The patient showed no significant medical findings. Intraoral examination (Figure 1) showed poor oral hygiene, multiple deep caries in permanent teeth, multiple remaining roots of primary teeth, and retained primary upper right canine. A palatal bulge was detected upon palpation on the right side of the anterior part of the maxilla.

Investigation and diagnosis

**Figure 2:**
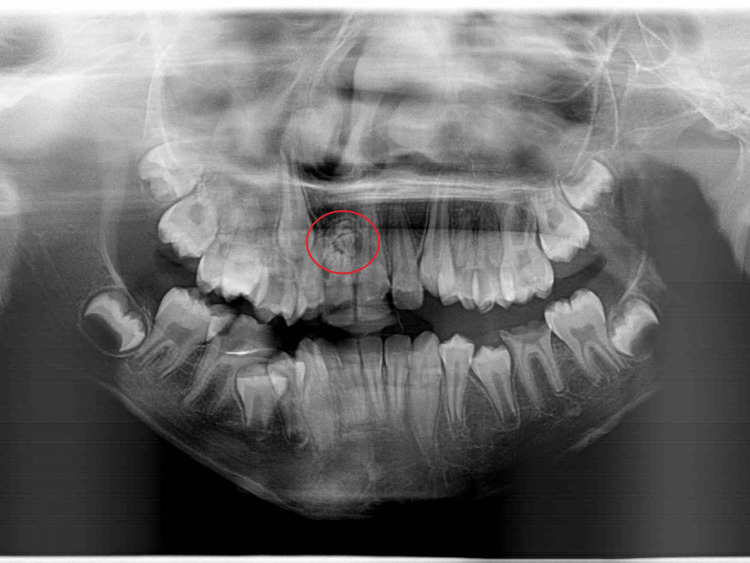
Panoramic radiograph The image shows a well-defined radiopaque lesion surrounded by a thin halo.

**Figure 3:**
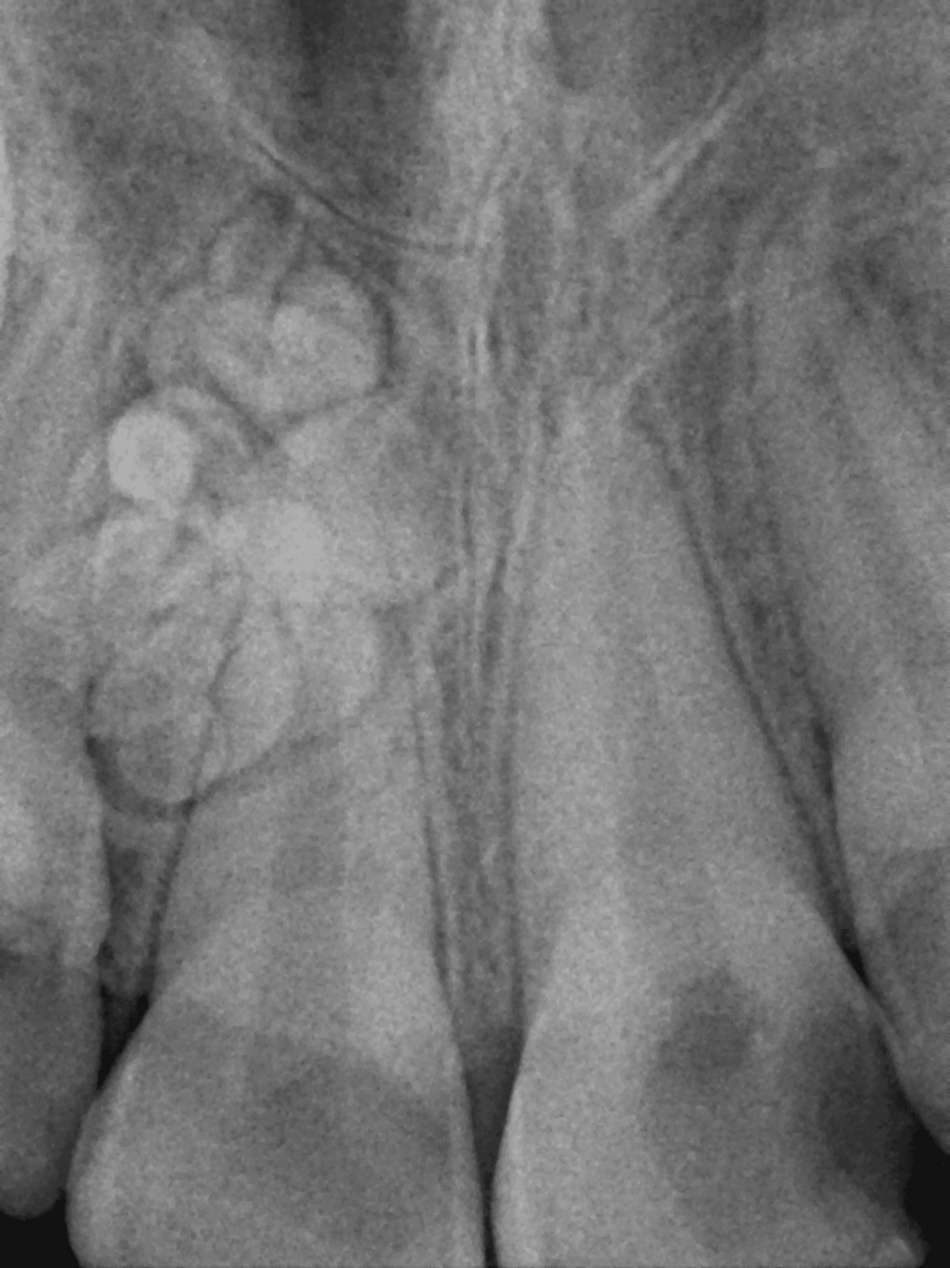
Periapical radiograph The radiograph shows multiple tiny tooth-like structures related to the right central incisor.

**Figure 4:**
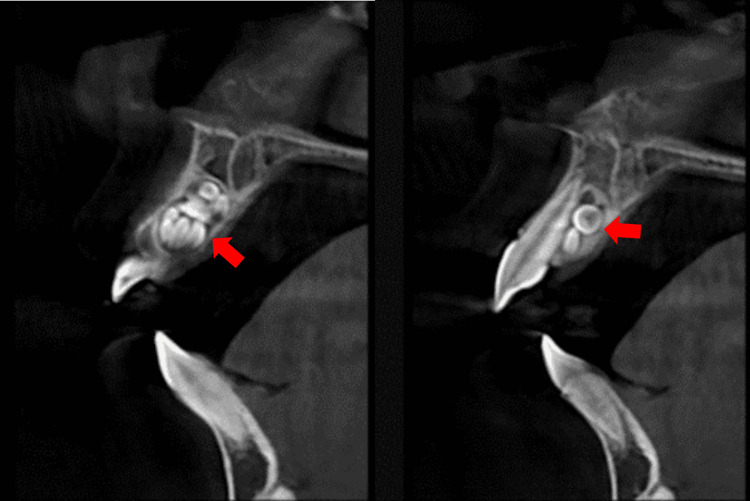
Cone beam computed tomography (axial view) The cone beam computed tomography image shows a palatially located lesion.

Panoramic radiograph (Figure 2) showed a well-defined radiopaque mass surrounded by a thin halo in the right maxillary anterior region. This mass comprised multiple small, tooth-like structures between the right permanent central and lateral incisors. Further investigation methods were performed, including a periapical radiograph (PA; Figure 3) and a cone beam computed tomography (CBCT) scan (Figure 4). These methods revealed the palatal location and extension of the tumor. The initial diagnosis of the lesion was compound odontoma according to the clinical and radiographic findings.

Treatment

**Figure 5:**
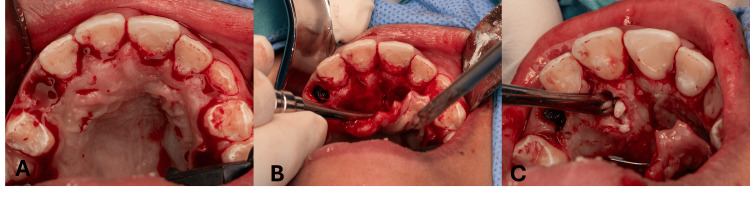
Surgical intervention Surgical incision (A), flap raising, and bone were exposed (B), and compound odontoma is exposed after osteotomy (C).

**Figure 6:**
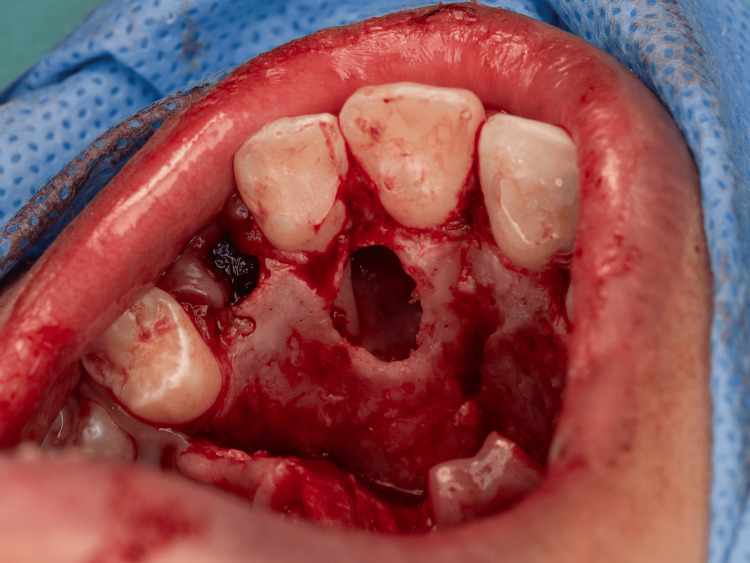
Intraoperative image An image after the lesion was removed showing an empty cavity with exposure of the lateral incisor root.

**Figure 7:**
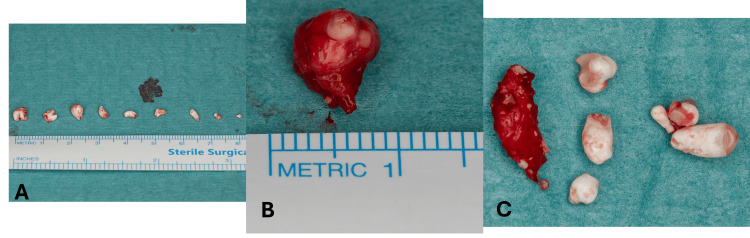
Compound odontoma Nine tiny tooth-like structures were extracted separately (A), a cystic granulation contains multiple odontomas (B), and cyst’s content (C).

**Figure 8:**
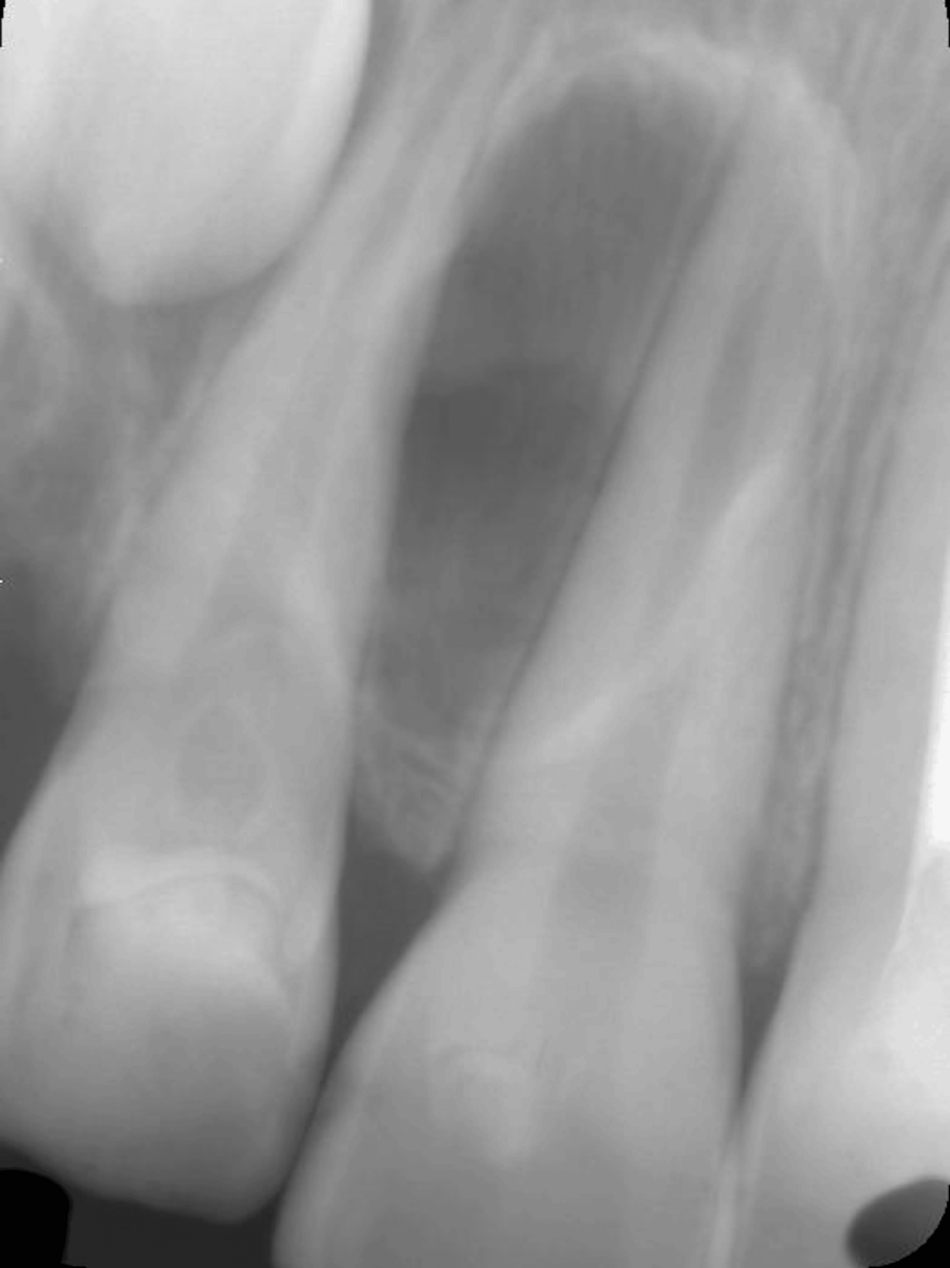
Periapical radiograph A periapical radiograph obtained immediately after lesion removal, showing the empty cavity.

**Figure 9:**
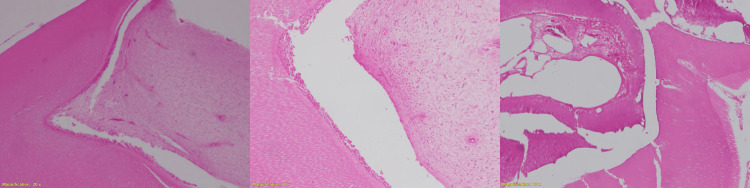
Histopathology The sample under a microscope revealed dental hard tissue and mesenchyme, distributed in dense fibrous tissue. It is formed of enamel matrix, mineralized dentin, and dental pulp.

Since the patient showed anxious dental behavior and needed comprehensive dental treatments, she was scheduled for surgical removal of the tumor and full mouth treatment under general anesthesia. Full mouth rehabilitation was done, and the conservative intraoral approach was carried out starting with an incision, then the flap was elevated (Figure 5), followed by osteotomy after bone removal; odontoma was exposed in addition to the exposure of the root of the right permanent lateral incisor (Figure 6). Inside the tumor, 15 tiny tooth-like structures were found; five pieces were inside a cyst (Figure 7). PA was taken before suturing (Figure 8) to ensure that all odontoma was removed, showing an empty cavity after mass removal. The excised tissues were sent for histo-pathological examination, and the results are shown in (Figure 9), confirming our initial diagnosis.

Follow-up

**Figure 10:**
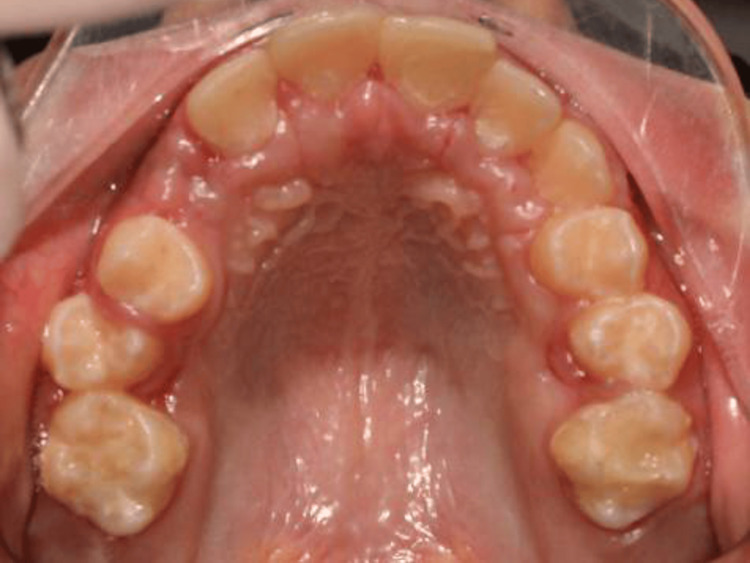
Clinical photograph two weeks follow-up Two weeks follow-up image shows healing.

**Figure 11:**
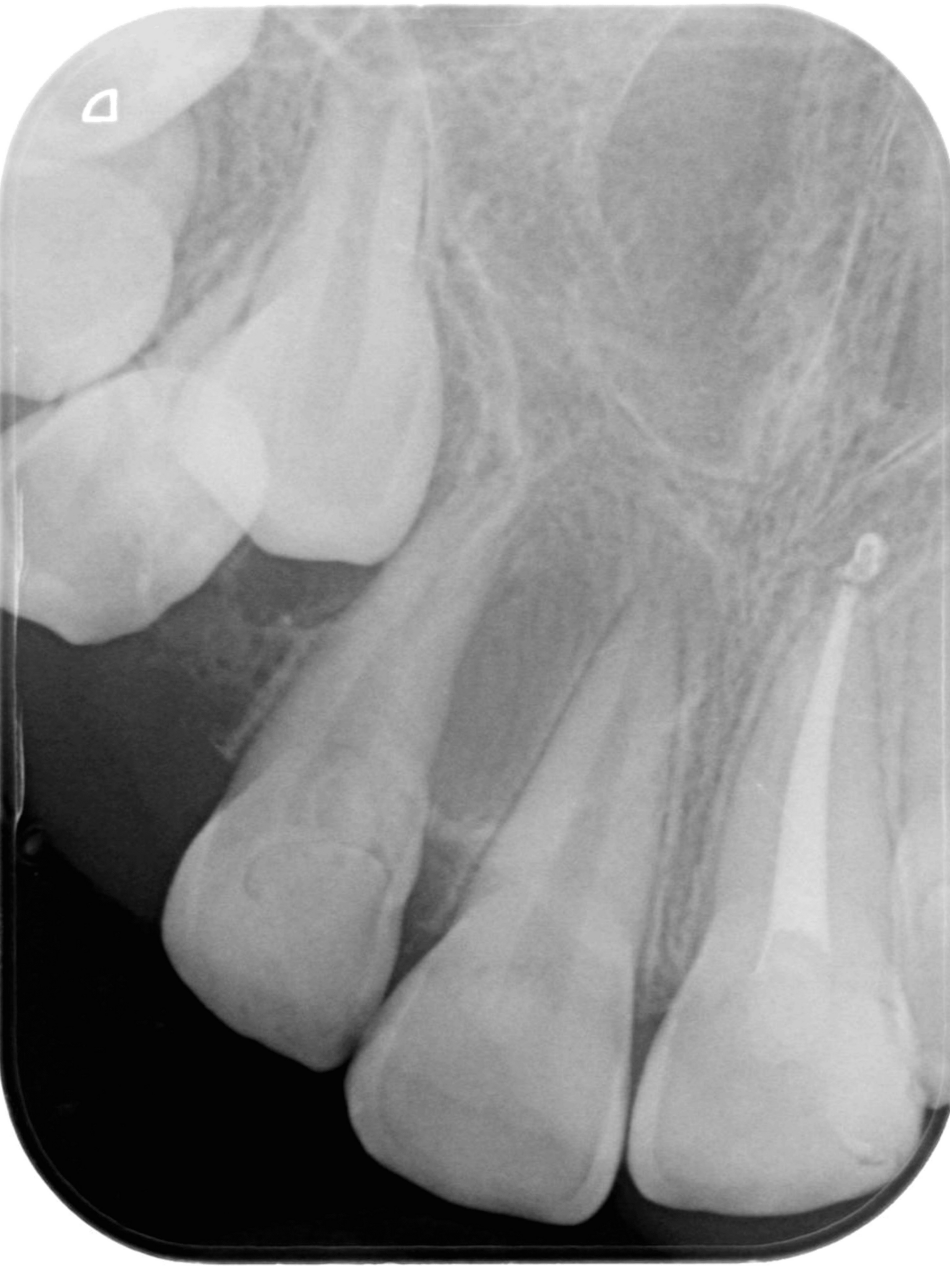
Periapical radiograph At two weeks of follow-up.

**Figure 12:**
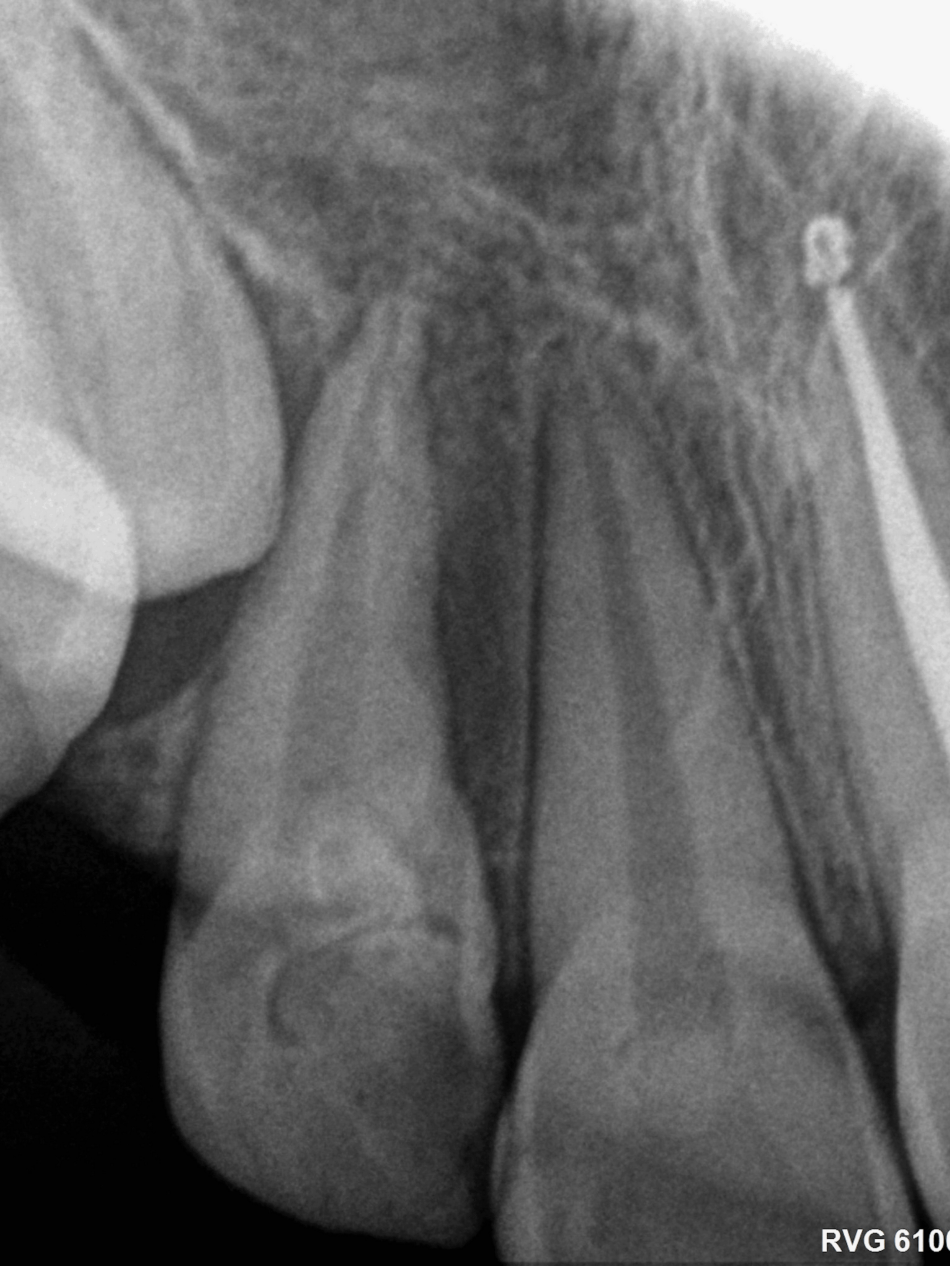
Periapical radiograph Periapical radiograph obtained after one month showing good bone remodeling.

**Figure 13:**
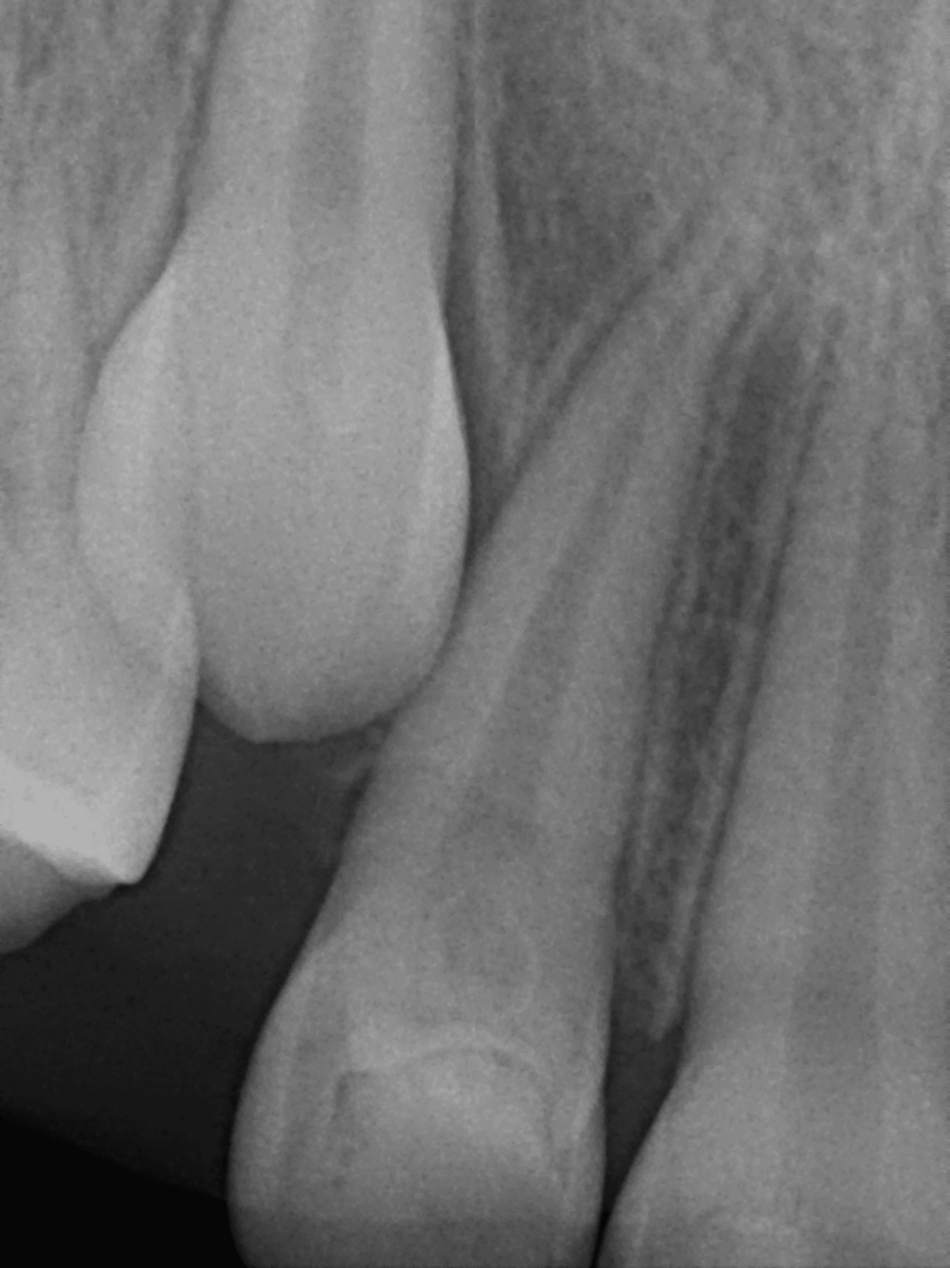
Periapical radiograph Obtained after six months of postoperative follow-up.

After two weeks, one month, and six months, follow-up was done postoperatively. The clinical photograph was taken (Figure 10), and bone remodeling was shown in PA (Figures 11-13).

## Discussion

Odontomas are the most common type of benign odontogenic tumors, representing around 70% of all odontogenic tumors of the jaws [1]. Compared to complex odontomas, compound odontomas occur more frequently in the general population [6,8] and are often diagnosed earlier than complex odontomas [6,8]. There is no known cause for the odontoma [2,7]. However, it has been suggested that infection and trauma at the site of the lesion are predisposing factors [7]. Compound odontomas are mainly located in the upper maxilla, either between the roots of erupted teeth or above the crowns of unerupted teeth [6,8]. Studies revealed that there is no difference in gender predisposing [6,9]. A study that analyzed 396 cases of compound odontomas showed that compound odontomas are generally diagnosed between 11 and 15 years [10]. Most odontomas are asymptomatic and found on routine radiographic examination [3,6]. However, some clinical manifestations were reported, mostly retention of permanent teeth and swelling [6]. Frequently, odontomas are associated with unerupted tooth [10]. Canines are the most commonly impacted tooth by odontoma, followed by upper central incisors and then third molars [11]. Radiographically, compound odontomas appear unilocular and contain multiple calcified mini tooth-like structures surrounded by a radiolucent zone [12]. The initial diagnosis of compound odontomas depends on the radiographic and clinical manifestations [3]. The histological assessment of odontomas frequently shows cementum, dentin, pulp tissue, and enamel matrix [13]. Histological examination regularly confirms the definitive diagnosis of compound odontomas [14]. Odontomas must be removed surgically to prevent possible conversion to odontoameloblastoma and cyst formation [15]. In some cases, a bone graft is needed after tumor removal, depending on the odontoma's size and location [5]. The current patient is pediatric, and the bone remodeling rate in children is three times higher than in adults [16]; therefore, there is no need to place a bone graft at the site of the tumor. The recurrence of an enucleated odontoma is rare; however, young children require long-term follow-up [5].

## Conclusions

This case report discusses the surgical removal of the tumor, emphasizes the importance of early diagnosis, and encourages surgical intervention to manage compound odontomas and avoid the tumor's unwanted consequences.

## References

[1] Levi-Duque F, Ardila CM (2019). Association between odontoma size, age and gender: multivariate analysis of retrospective data. J Clin Exp Dent.

[2] Khalifa C, Omami M, Garma M, Slim A, Sioud S, Selmi J (2022). Compound-complex odontoma: a rare case report. Clin Case Rep.

[3] Ćabov T, Fuchs PN, Zulijani A, Ćabov Ercegović L, Marelić S (2021). Odontomas: pediatric case report and review of the literature. Acta Clin Croat.

[4] Soluk-Tekkeşin M, Wright JM (2018). The World Health Organization classification of odontogenic lesions: a summary of the changes of the 2017 (4th) edition. Turk Patoloji Derg.

[5] Zidane FE, Azzouz Y, Fawzi R (2022). Surgical management of compound odontoma associated with unerupted tooth: a case report. Pan Afr Med J.

[6] Hidalgo-Sánchez O, Leco-Berrocal MI, Martínez-González JM (2008). Metaanalysis of the epidemiology and clinical manifestations of odontomas. Med Oral Patol Oral Cir Bucal.

[7] López-Areal L, Silvestre Donat F, Gil Lozano J (1992). Compound odontoma erupting in the mouth: 4-year follow-up of a clinical case. J Oral Pathol Med.

[8] Soluk Tekkesin M, Pehlivan S, Olgac V, Aksakallı N, Alatli C (2012). Clinical and histopathological investigation of odontomas: review of the literature and presentation of 160 cases. J Oral Maxillofac Surg.

[9] Fernandes AM, Duarte EC, Pimenta FJ, Souza LN, Santos VR, Mesquita RA, de Aguiar MC (2005). Odontogenic tumors: a study of 340 cases in a Brazilian population. J Oral Pathol Med.

[10] Shulman ER, Corio RL (1987). Delayed eruption associated with an odontoma. ASDC J Dent Child.

[11] Katz RW (1989). An analysis of compound and complex odontomas. ASDC J Dent Child.

[12] Tomizawa M, Otsuka Y, Noda T (2005). Clinical observations of odontomas in Japanese children: 39 cases including one recurrent case. Int J Paediatr Dent.

[13] Lone PA, Kour I, Gandral A (2014). Intra oral approach for complex & compound odontomas (large or small). Modern Plastic Surgery.

[14] Maltagliati A, Ugolini A, Crippa R, Farronato M, Paglia M, Blasi S, Angiero F (2020). Complex odontoma at the upper right maxilla: surgical management and histomorphological profile. Eur J Paediatr Dent.

[15] Neville BW, Damm DD, Allen CM, Chi AC (2015). Oral and Maxillofacial Pathology (E-book). https://books.google.com.sa/books?id=DmVgDwAAQBAJ&lpg=PP1&ots=9HYW2A693f&dq=Oral%20and%20Maxillofacial%20Pathology%203rd%20Ed&lr&hl=ar&pg=PP1#v=onepage&q=Oral%20and%20Maxillofacial%20Pathology%203rd%20Ed&f=false.

[16] Parfitt AM, Travers R, Rauch F (2000). Structural and cellular changes during bone growth in healthy children. Bone.

